# Current State and Advances in Antimicrobial Strategies for Burn Wound Dressings: From Metal-Based Antimicrobials and Natural Bioactive Agents to Future Perspectives

**DOI:** 10.3390/ijms26094381

**Published:** 2025-05-05

**Authors:** Andrea Osmokrovic, Jasmina Stojkovska, Tanja Krunic, Predrag Petrovic, Vesna Lazic, Jovana Zvicer

**Affiliations:** 1 Faculty of Technology and Metallurgy, University of Belgrade, Karnegijeva 4, 11000 Belgrade, Serbia; 2 Innovation Center of Faculty of Technology and Metallurgy, University of Belgrade, Karnegijeva 4, 11000 Belgrade, Serbia; 3 Faculty of Agriculture, University of Belgrade, Nemanjina 6, 11080 Belgrade, Serbia

**Keywords:** antimicrobial resistance, biofilm, infection, topical therapy, alternative antimicrobials

## Abstract

Burn wounds represent a complex clinical challenge, primarily due to their high susceptibility to infections and the frequent formation of the biofilm, which significantly hinder the healing process. Therefore, effective infection prevention and management are critical components of burn wound care. This review provides a comprehensive overview of the current and emerging antimicrobial strategies in burn management, with a particular focus on alternative approaches to conventional antiseptics and antibiotics. This manuscript highlights the role of metals and metal-based agents, including silver, zinc oxide, and copper compounds, alongside plant-derived bioactive substances such as aloe vera, marigold, and turmeric. Additionally, the potential of antimicrobial peptides and probiotics as innovative therapeutic options is explored, emphasizing their antimicrobial, anti-inflammatory, and pro-healing properties. Finally, this review presents an analysis of recent patents in the field of burn wound care, offering insights into current trends and future directions in the development of advanced wound dressings. By addressing both established and novel strategies, this review aims to provide a valuable resource for clinicians, researchers, and innovators seeking to improve outcomes in burn wound management.

## 1. Introduction

**Figure 1:**
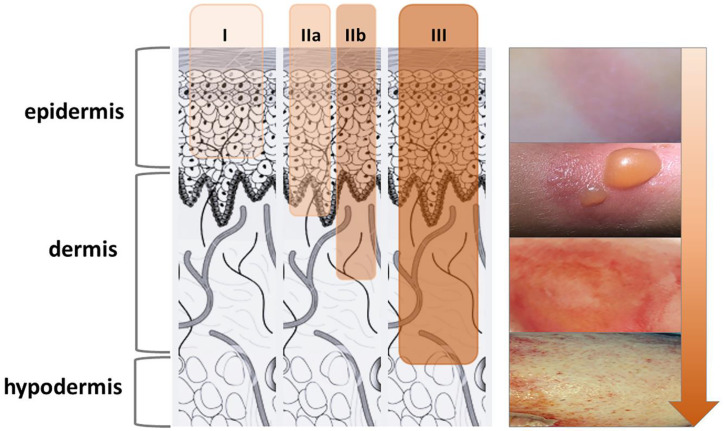
First-, second-, and third-degree burns.

Burns are a severely debilitating class of wounds that are most commonly caused by heat (about 90% of all burns) but also by radiation, radioactivity, electricity, friction, or contact with chemicals. In addition to skin injury, deeper structures may be affected depending on the duration and intensity of the harmful agent. Burn wounds are localized tissue damage, but in severe cases, they can lead to systemic disorders (e.g., toxic shock, multiple organ dysfunction syndrome) that may endanger a patient’s life or have a lifelong health impact. Annually, around 180,000 deaths worldwide are attributed to burns, with the majority occurring in low- and middle-income countries [1]. Moreover, the burn death rate in children is over seven times higher in these regions than in high-income countries [1]. Globally, direct burn care costs and management vary widely but tend to be expensive, thus placing an enormous burden on healthcare systems. For example, the UK’s National Health Service has estimated to spend ₤5.3 billion pounds annually on burn wound care and management [2]. Burns are classified as first-, second-, or third-degree depending on how deep and severe the skin’s surface is penetrated (Figure 1).

First-degree or superficial burns affect only the epidermis, the outer layer of the skin (e.g., mild sunburn). Spontaneous healing occurs in a short period of time (a couple of days), while long-term tissue damage is rare and usually consists of an increase or decrease in skin color. Second-degree or partial-thickness burns are caused by the more intense effect of the harmful agent and involve damage to the epidermis and part of the dermis layer of the skin. Second-degree burns can be differentiated into superficial (IIa) and deep (IIb) based on the depth of dermal damage. They are prone to infection, but with proper prevention, epithelization may occur spontaneously. However, in deeper dermis injuries, this process takes longer (up to several weeks) and results in scarring due to fibrous changes in the dermis. In addition, deep second-degree burns may progress to third-degree burns due to infection and the subsequent destruction of the entire dermis. Third-degree or full-thickness burns involve the destruction of the entire epidermis and dermis, with the loss of most skin functions. They may also include damage to the underlying fat, muscles, tendons, and even bones. With this degree of damage, there is no possibility of spontaneous healing without medical intervention (e.g., skin grafts) unless the lesion is up to 1% of the total body surface. Even then, healing takes a very long time (several months) and results in excessive scarring. Apart from the degree of the burn, the patient’s prognosis also depends on the affected surface area, specific anatomic sites, medical history, and age of the patient.

## 2. Burns and Infection

When the skin is injured, its function as a physical barrier against various agents, including microorganisms (bacteria, viruses, and fungi), is compromised, significantly increasing the risk of infection. In addition, the deeper the burn, the greater the risk of bacteremia and sepsis, as pathogens can penetrate the circulatory system more easily. Patients with severe burns are immunosuppressed; consequently, strict infection control practices are essential in burn care units. Indeed, infection (including cellulitis, graft loss, and sepsis) is one of the biggest challenges, often being the most common complication that occurs in over 20% of burn patients despite optimal therapy (e.g., topical antimicrobials, antibiotic stewardship, early and aggressive medical interventions like debridement, grafting, etc.) [3]. Furthermore, it is the leading cause of death in 75% of all burn-related deaths [2].

As expected, there is a high correlation between the presence of a large number of bacterial cells in the wound (>10^5^ CFU/g tissue) and signs such as pain, edema, wound deterioration, and even sepsis. Burn wounds are mostly colonized by opportunistic pathogens such as *Pseudomonas aeruginosa*, *Acinetobacter baumannii*, and *Staphylococcus aureus* [2], which are commonly found in hospital environments. The ability of these pathogens to form biofilms is a major contributor to their pathogenic success and the considerable complications they cause in burn wound management. Biofilm is a structured community of millions of microbial cells encased in a self-produced extracellular matrix adherent to the wound surfaces [4] and is estimated to be involved in over 60% of all wound infections [5]. The extracellular matrix, which constitutes 75–90% of the biofilm, protects bacteria from antibiotics and the immune system, contributing to their resistance. In addition, bacterial cells within the biofilm exhibit altered phenotypes compared to their planktonic counterparts. They exist in a reduced metabolic state, making them highly tolerant to conventional antimicrobial agents and difficult to eradicate. This tolerance enables them to withstand prolonged exposure to both local and systemic antibiotic treatments without losing viability, as biofilms have been estimated to be 10 to 1000 times more resistant to antibiotic treatment than planktonic bacteria [6,7,8,9].

In addition, the overuse and/or misuse of antibiotics in recent decades has created selective pressure and highly accelerated the spread of antibiotic-resistant genes among pathogenic species of bacteria. This has ultimately led to the global public health crisis we are facing today. Unfortunately, antimicrobial resistance (AMR) disproportionately affects immunosuppressed patients, such as those with serious burns or cancer. A recent study published in The Lancet using predictive statistical modeling approximated that in 2019, there were 4.95 million deaths worldwide associated with antibiotic-resistant bacteria, of which approximately 25% were directly attributed to AMR [10]. Moreover, the use of different antibiotics often leads to the accumulation of resistance genes in pathogenic bacteria, driving their evolution toward multidrug-resistant (MDR) and even pan-drug-resistant (PDR) phenotypes, for which no effective treatments are currently available. These growing challenges highlight the urgent clinical need for the development of novel antibiotics and new strategies for local infection control. Unfortunately, the development of new antibiotics remains slow due to high costs and low profitability, shifting the focus toward alternative antimicrobial agents.

Conventional treatment of infected wounds and those at high risk, such as burns, relies heavily on systemic antibiotics and topical antiseptic solutions. However, in burn injuries, compromised local microcirculation often necessitates increased systemic antibiotic doses to achieve therapeutic concentrations at the wound site, thereby raising concerns about systemic toxicity. Meanwhile, topically applied antiseptics are often rapidly inactivated by the abundant wound exudates typically observed in second-degree burns. In this context, wound dressings impregnated or loaded with antimicrobial agents have emerged as valuable tools in clinical practice, enabling sustained and localized delivery of therapeutic agents directly to the wound site. The following section discusses the advantages and limitations of commonly used antimicrobial wound dressings for treating burn injuries.

## 3. Antimicrobial Wound Dressings: Traditional vs. Advanced

A diverse range of antimicrobial wound dressings, both traditional and advanced, are currently available for the treatment of infected wounds and those at high risk of infection, such as burns. Grand View Research reported that the global antimicrobial wound dressing market generated a revenue of approximately USD 1500.10 million in 2023, with a predicted compound annual growth rate (CAGR) of 6.6% from 2024 to 2030, reaching an estimated USD 2188.8 million by 2030 [11]. Traditional wound dressings, based on impregnated gauzes and non-woven sponges, ropes, or strips saturated with various compounds, are still widely used worldwide owing to their affordability. These dressings are mostly impregnated with mineral oils (petroleum gel or petroleum jelly) and are characterized by porous, greasy, and non-adhesive structures that help minimize damage to the newly formed granulation tissue during removal. Their antimicrobial activity is usually based on antibiotics (e.g., fusidic acid, framycetin, silver sulfadiazine (SSD)) or conventional antiseptics (e.g., povidone−iodine, chlorhexidine, polyhexamethylene biguanide (PHMB), bismuth tribromophenate), while some are impregnated with medical-grade honey. However, owing to their hydrophobicity, traditional dressings do not absorb wound secretions, and a secondary dressing is always required.

On the other hand, advanced antimicrobial wound dressings are created to maintain optimal healing conditions, like a physiologically moist environment that supports re-epithelialization, reduces pain, and minimizes scarring, which is even more beneficial in burn wounds due to their unique challenges. Advanced antimicrobial dressings are generally based on natural and/or synthetic polymers, often functionalized with antimicrobial additives, either through incorporation or surface coating. Natural polymers (e.g., alginate, collagen, chitosan, cellulose, gelatin, and carboxymethylcellulose) are often considered superior to synthetic polymers due to their advantages for cellular growth and wound healing, such as biocompatibility, biodegradability, and ability to mimic the extracellular matrix (ECM). Recently, silk fibroin has gained significant attention [12] due to its promotion of rapid and uncomplicated scarring, which is beneficial in the treatment of burns, especially in the region of the face or hands. Conversely, synthetic polymers (e.g., polyurethane, polyethylene glycol, and silicon) are preferred due to their customizable properties, reproducibility, superior mechanical properties, and longer shelf life [13]. The combination of natural and synthetic polymers has led to the emergence of composite dressings, which dominate the current market due to their improved structural integrity and superior moisture-handling capacity. Despite the wide variety of available products, there is still no consensus on the optimal dressing for burn wound coverage. Given the rising awareness of AMR, modern antimicrobial strategies are increasingly based on non-antibiotic agents, such as silver and honey, and conventional antiseptics like iodine compounds, chlorhexidine, or PHMB, which help prevent infection while reducing the risk of resistance development.

**Figure 2:**
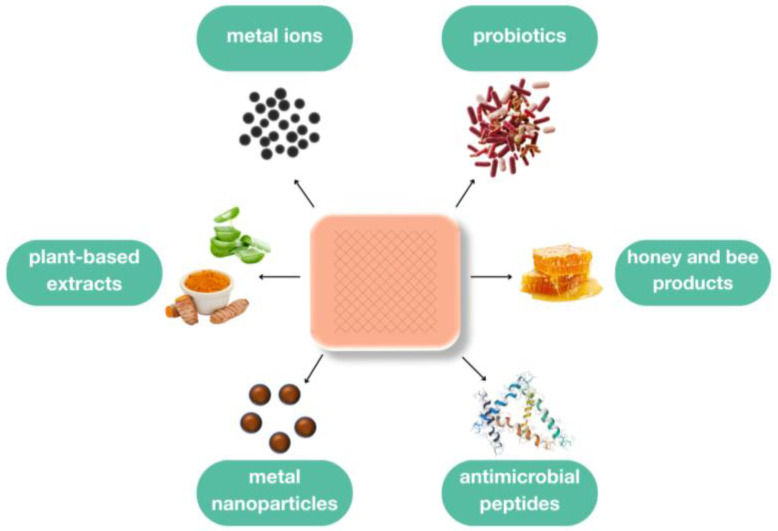
The scheme of active components in the burn wound dressings.

This review provides a comprehensive overview of the current state and future perspectives of antimicrobial agents incorporated into wound dressings aimed at burn management (Figure 2). Conventional antiseptics have been excluded due to their well-documented efficacy and clinical applications in the literature. Instead, this review focuses on the advantages and disadvantages of alternative antimicrobials and emerging strategies that require further investigation to better understand their potential roles in burn wound management.

## 4. Antimicrobial Agents in Wound Dressings

The revolutionary discovery of antibiotics in the early 20th century with the introduction of penicillin into clinical practice led to the complete loss of interest in other types of antibacterial agents, and only with the emergence of AMR was this interest renewed. Unlike antibiotics, which target specific bacterial processes, such as cell wall synthesis (e.g., β-lactams), protein synthesis (e.g., tetracyclines), DNA/RNA replication (e.g., fluoroquinolones), or metabolic pathways (e.g., sulfonamides), alternative antimicrobials exhibit nonspecific and multi-targeted modes of action and consequently have a lower likelihood of resistance development. They are often effective against both Gram-positive and Gram-negative bacteria, including antibiotic-resistant strains. In addition, some may exhibit other benefits, such as anti-inflammatory, antioxidant, and wound-healing properties. However, depending on their mechanism of action, some may also affect eukaryotic cells, including those of humans.

The following sections provide an overview of the key classes of alternative antimicrobial agents used in wound dressings, with a particular focus on metal-based compounds, natural bioactive agents, and innovative biological strategies.

### 4.1. Metals and Metal-Based Antimicrobials

#### 4.1.1. Silver

Silver has been widely used in burn wound treatment in various formulations, ranging from simple solutions (e.g., 0.5% silver nitrate) and creams/ointments (e.g., 1% SSD, a complex of silver with the antibiotic sulfadiazine) to more complex traditional (e.g., SSD-impregnated dressing) and advanced dressings (e.g., silver-coated or silver-embedded wound dressing). Despite being introduced in the late 1960s, SSD cream remains the standard of care in many burn centers despite ongoing debates regarding its efficacy and potential drawbacks. The biologically active form of silver appears to be its cationic species (Ag⁺) [14], which exhibits potent antimicrobial properties through multiple modes of action that have only recently been fully elucidated. These include (i) interaction with peptidoglycan in the bacterial cell wall and membrane, leading to pore formation and structural damage; (ii) disruption of electron transport followed by inhibition of cellular respiration; (iii) inhibition of DNA replication; and (iv) induction of oxidative stress through the generation of reactive oxygen species (ROS) [15]. According to a 2023 Data Bridge Market Research report, silver wound products represent a dominant share of the global antimicrobial wound care market, with a value of USD 990.05 million in 2022 and a projected CAGR of 8.2% until 2030 [16]. Although silver has been shown to be effective in infection control, the available in vitro and clinical evidence regarding its efficacy in accelerating wound healing remains inconsistent and contradictory [17]. Nevertheless, the widespread use of these products across medical disciplines and their incorporation in a wide range of consumer products (e.g., toothbrushes, pastes, water filters, food storage containers, and clothes) have contributed to the emergence of clinically significant silver-resistant [18,19,20,21]. This underscores the urgent need for rigorous antimicrobial stewardship across all antimicrobial agents, not just for antibiotics.

#### 4.1.2. Zinc

Zinc is an essential trace element that has been used in medical applications in the form of zinc oxide (ZnO) as a mild antimicrobial that exhibits its activity through several mechanisms of action, including (i) membrane disruption, (ii) production of ROS, and (iii) release of toxic Zn^2+^ ions that cause mis-metalation of enzymes and disruption of ionic homeostasis [22]. It is commonly used in topical treatments in the form of creams/ointments or impregnated traditional dressings. Double-blind clinical studies demonstrated its efficacy in *S. aureus*-infected excisional wounds and showed that topically applied ZnO not only significantly reduced bacterial growth but also the use of antibiotics in the treatment group. [23]. In contrast, advanced zinc-based dressings, designed for use on exuding wounds such as burns, primarily rely on the controlled release of zinc ions from hydrogel matrices. Specifically, zinc ions disrupt cell membranes by activating peptidoglycan autolysins, causing oxidative stress and DNA damage in Gram-positive *Staphylococcus aureus* and Gram-negative *Escherichia coli* [24]. Even though zinc ions are less potent than silver in terms of antimicrobial activity, they contribute significantly to wound healing by exerting antioxidant and anti-inflammatory activities, promoting granulation tissue formation, stimulating collagen synthesis, and enhancing keratinocyte proliferation and migration during wound repair [25]. However, bacteria can develop mechanisms to tolerate high levels of zinc (e.g., reduced uptake, efficient efflux, internal or external sequestration, and transformation to less toxic forms). In addition, higher bacterial tolerance to zinc ions can be connected to their resistance to certain antimicrobials (e.g., sulphametoxasol, ciprofloxacin) or acquired multidrug resistance (e.g., carbapenems, third-generation cephalosporins), as zinc and antibiotic resistance genes are often found on the same plasmid [26]. This suggests that heavy metals, like zinc, may co-select for antibiotic resistance traits in bacterial populations [26,27], which could also be of relevance in everyday life, as Zn compounds are widely used in ointments and skin preparations aimed to be used from a very early age [28].

#### 4.1.3. Copper

Copper is also an essential trace element that demonstrates efficacy against various bacterial strains, including resistant strains, through multiple antibacterial mechanisms: (i) damage to membrane phospholipids, resulting in the loss of membrane integrity and cell death; (ii) triggering of ROS production, leading to the rest of cell respiration and DNA breakdown; and (iii) inactivation of iron–sulfur cluster in enzymes, followed by damage to the central catabolic and biosynthetic pathways [29]. Beyond its strong antimicrobial properties, copper plays an important role at every stage of the wound-healing process, including skin regeneration and angiogenesis [30], which zinc and silver may not support to the same extent. In particular, the copper-plasma peptide complex stimulates wound healing by promoting the production of ECM components, such as collagen, proteoglycans, glycosaminoglycans, and chondroitin sulfate. At the same time, it induces the expression of metalloproteinases and antiproteases, which contribute to the degradation of damaged proteins, leading to tissue remodeling [31]. In addition, copper increases the expression of vascular endothelial growth factor (VEGF), thus significantly accelerating wound closure and improving the quality of the regenerating tissue [32]. The first advanced wound dressings impregnated with copper in the form of copper oxide microparticles received FDA and CE clearance in 2018 for the treatment of various wounds, including first- and second-degree burns [33]. However, similar to other metals, copper may lead to the development of higher bacterial tolerance, with the horizontal transfer of copper resistance genes observed in *S. aureus* and *L. monocytogenes* [34]. Recently, it was found that the copper and silver resistance genes form a cluster called the “copper homeostasis and silver resistance island” [35]. Furthermore, copper like other metals may also co-select for antibiotic resistance, including resistance to last-resort antibiotics such as colistin [35], thereby contributing to the emergence and spread of AMR.

#### 4.1.4. Metal Nanoparticles

Numerous research groups are actively engaged in the synthesis and development of various metal nanoparticles (NPs), including silver (AgNPs), copper (CuNPs), gold (AuNPs), and metal oxide nanoparticles such as zinc oxide (ZnO NPs), and copper oxide (CuO NPs) [36,37,38,39,40,41,42]. This growing interest is primarily attributed to their superior antimicrobial activity compared to their bulk and ionic counterparts [43,44,45]. In addition, nanoparticles often exhibit a multi-targeted action against not only bacteria but also biofilms, which conventional antibiotics often fail to penetrate. Their antimicrobial activity is closely linked to key physicochemical properties, such as size, shape, and surface charge [46,47,48]. Specifically, smaller nanoparticles demonstrate enhanced antimicrobial and anti-biofilm activities compared to larger nanoparticles, and spherical nanoparticles tend to be more effective than triangular or rod-shaped nanoparticles [46,49]. This can be attributed to their larger surface-area-to-volume ratios, which facilitate a higher release rate of metal ions and promote stronger interactions with bacterial cells, including easier penetration of the bacterial membrane [48,50]. Moreover, positively charged nanoparticles exhibit greater bactericidal activity than neutral or negatively charged particles, a phenomenon that can be explained by the negatively charged nature of bacterial cell membranes, which promotes electrostatic interactions [47].

Electrochemically synthesized AgNPs in alginate colloid solution and those immobilized in alginate microfibers have been shown to enhance the healing process in a rat model of deep second-degree burns [42]. These AgNPs exhibited similar effects on burn wound healing as the two control commercial products (1% SSD cream and Ca-alginate dressing containing Ag ions) but at a significantly lower concentration of silver (up to 100-fold). Similarly, biosynthesized ZnO NPs (0.2%) from *Arthrospira maxima* exhibited comparable positive effects on the healing of burns infected with *A. baumannii* in a rat model, as did colistin, a last-resort polymyxin antibiotic, used for treatment of resistant Gram-negative infections [51]. Given that resistant Gram-negative pathogens, including *A. baumannii*, are classified by the World Health Organization as priority 1 (critical) for the development of new therapeutic options, this approach offers a promising alternative that may help mitigate the emergence of AMR by reducing the reliance on antibiotics. Biosynthesized CuO NPs (0.2%, ~35 nm in diameter) also demonstrated significant improvements in second-degree burn treatment in a mouse model, particularly in re-epithelization, collagen deposition, and angiogenesis, compared to the untreated control group [52]. Similar positive effects were observed with AuNPs and CuNPs (1 μM, ~20 nm in diameter) in wound treatment in a rat model [37,53,54]. Recently, research has advanced toward the development of novel antimicrobial agents that combine metal nanoparticles with conventional antibiotics to enhance antimicrobial efficacy through the synergistic effects between these two components [55,56]. For example, AgNPs functionalized with ampicillin exhibited stronger antimicrobial effects against *E. coli.* than pure AgNPs. In addition, Ag-Au NPs with doxycycline displayed enhanced antimicrobial activity against *Pseudomonas aeruginosa*, *Escherichia coli*, *Staphylococcus aureus*, and *Micrococcus luteus* compared to pure Ag-Au NPs [57].

Even though metal nanoparticles have shown various positive effects on wound healing, their clinical application raises significant concerns and faces regulatory challenges due to their stability [58], cytotoxicity [59], accumulation [60], long-term safety, and lack of standardized testing protocols [60,61].

### 4.2. Bee Products

#### 4.2.1. Honey

Honey has been used since ancient times for the treatment of wounds, especially burns; however, its beneficial effects on the healing process have only recently been understood. Honey is known to exhibit various positive effects due to its antimicrobial and anti-biofilm activities [62], as well as its anti-inflammatory [63,64] and immunomodulatory properties [65]. It is widely used in various forms, from pure honey gel and traditional honey-impregnated gauzes to advanced polymer-based wound dressings coated with honey. In 2024, Grand View Research reported that the global honey dressing market size was valued at approximately USD 33.72 million in 2023, with a projected CAGR of 4.91% from 2024 to 2030 [66]. The dominant position in the market is firmly held by manuka honey, but there are also dressings infused with other types of medical-grade honey (chestnut and buckwheat), while other types (e.g., acacia honey) are being intensively investigated [67,68]. Their antimicrobial activity primarily relies on (i) the enzymatic production of hydrogen peroxide through the oxidation of glucose, which disrupts the membrane; (ii) the inhibition of protein synthesis and enzyme activity; (iii) the promotion of nucleoside oxidation; and (iv) the impairment of energy production [69]. In addition, its low pH (3.2–4.5) inhibits microbial growth, while the high sugar content induces osmotic pressure, leading to efflux of water from cells, followed by dehydration [70]. Even though there is significant variation in the properties of various types [71], honey resistance has not yet been observed [72,73]. To date, honey-based dressings have only been used for the healing of limited superficial partial-thickness wounds, while the safety, efficacy, and use for the treatment of more severe, extensive, or complex burn wounds have not yet been established. In addition, these dressings are sticky and adhere to the wound surface, which may be uncomfortable for patients during removal. The next generation of honey-based wound dressings is moving toward the development of new formulations that incorporate additional antimicrobial compounds, either conventional (e.g., antibiotics [74] and antiseptics [75]) or nonconventional (e.g., silver nanoparticles [76], bioactive glass [77], and propolis [78]).

#### 4.2.2. Propolis

Propolis is a resinous substance produced by bees and is composed of beeswax, salivary secretions, and plant exudates. Its chemical composition can vary significantly depending on its geographic and botanical origins, which in turn influences its biological activity. Propolis is notably rich in polyphenols, including pharmacologically active phenylpropanoids, such as caffeic acid phenethyl ester (CAPE) and artepillin C, as well as flavonoids, like chrysin and galangin. These compounds contribute to their well-documented antioxidant, antibacterial, and anti-inflammatory properties, all of which may support wound-healing processes [79,80]. Although clinical data on the use of propolis in human burn wounds remain limited, numerous animal studies have demonstrated its therapeutic potential in treating burns. For instance, propolis-treated burn wounds were reported to exhibit approximately 50% thicker granulation tissue in the wound center than SSD-treated counterparts [81]. Additionally, the clinical signs of inflammation, such as erythema, warmth, and edema, were significantly reduced in the propolis group. Further animal studies have investigated the efficacy of propolis extracts in both infected and non-infected burn wounds [82,83]. Notably, treatment with a concentrated (100%) propolis extract reduced the total wound healing time by approximately 20% compared to that of the control (19–20 vs. 25 days). These studies also revealed a concentration-dependent effect on the shortening of both the inflammatory and proliferative phases of healing. In vitro analyses support these findings, showing that propolis extracts stimulate NO production in a dose-dependent manner and enhance the synthesis of extracellular matrix components, such as collagen, during the early stages of wound repair [79].

### 4.3. Plant-Based Compounds

In many regions of the world, particularly in developing countries, plant-based products play an important role in the management of burns [84]. Scientific evidence increasingly supports traditional knowledge regarding their therapeutic efficacy, with some plant-derived treatments demonstrating effects that may be comparable or even superior to those of conventional wound care methods. These natural remedies are rich in diverse phytochemicals and often exhibit multiple beneficial properties, including antiseptic, anti-inflammatory, and antioxidant effects, as well as additional activities, such as analgesic action, tissue regeneration, and inhibition of excessive scar formation. This multifunctionality is particularly advantageous for burn wound healing. The mechanisms of action of plant-based treatments typically involve the following: (i) modulation of inflammation through the inhibition of proinflammatory cytokines, such as interleukin (IL)-1β, IL-6, and tumor necrosis factor-alpha (TNF-α); (ii) suppression of neutrophil activity and reduction of reactive oxygen species (ROS) production; (iii) stimulation of fibroblast and keratinocyte proliferation; and (iv) promotion of neovascularization. These effects synergistically facilitate granulation tissue formation and epithelialization, thereby accelerating the healing of burn wounds [85].

The following section provides an overview of some of the most promising natural sources utilized in burn wound treatment, with evidence ranging from in vitro studies using keratinocytes and fibroblast cell lines to in vivo animal models and clinical trials. Despite extensive research on advanced wound dressings incorporating plant-derived bioactive compounds, commercially available plant-based wound care products remain largely limited to simple formulations, such as gels, creams, and ointments. This discrepancy arises from challenges in standardization, as plant extracts exhibit variability depending on the species, cultivation conditions, and extraction methods, making batch-to-batch consistency difficult. In addition, regulatory issues regarding advanced dressings often require extensive clinical validation. Stability issues, including the susceptibility of plant-derived compounds to degradation, further limit their integration into complex wound dressing formulations. Moreover, high production costs and slow industry adoption hinder the large-scale commercialization of such innovations. Until these challenges are addressed, simpler pharmaceutical formulations will continue to dominate the market for these products.

*Aloe barbadensis miller* (*Aloe vera*) is a widely cultivated succulent plant, and its bioactive components include mucilaginous polysaccharides, sterols, vitamins, and phenolic compounds. Increasing clinical evidence supports the traditional use of *A. vera* in burn wound management due to its hydrating and soothing properties, as well as its anti-inflammatory, antioxidant, and antimicrobial activities [86,87]. A systematic review of multiple clinical trials evaluating *A. vera*-based treatments for burns found that healing times were significantly shorter in *A. vera*-treated groups than in controls [88]. Furthermore, a recent study on patients with symmetrical second-degree burns (burn wounds on both arms) reported a significantly shorter healing duration in those treated with *A. vera* cream (0.5%) compared to those treated with the standard SSD cream (mean healing times of 15.9 vs. 18.7 days, respectively), with no observed wound infections in either group [89]. Animal studies have also demonstrated the efficacy of *A. vera* extract in burn treatment, not only when applied topically but also when administered orally, promoting enhanced tissue regeneration, vascularization, and improved antioxidant status in the body. However, as expected, topical application was more effective than oral administration [90].

*Calendula officinalis* (*marigold*) is a well-recognized medicinal plant, and its bioactive components include carotenoids, polyphenols, polysaccharides, and terpenoids. However, clinical research specifically evaluating the efficacy of *C. officinalis* in burn wound management is limited. A clinical study assessing the effectiveness of topical calendula ointment in patients with partial- and full-thickness burns found it to be as effective as standard debridement therapy while also resulting in lower pain perception and a reduced incidence of adverse effects [91]. In addition, oral supplementation with calendula extract, both in human patients with second-degree burns and in animal models, has demonstrated benefits as an adjunct to standard therapy, leading to enhanced recovery and accelerated wound healing [92,93].

*Hypericum perforatum* (*St. John’s wort*) is a well-known medicinal plant that contains, in addition to phenolic compounds such as flavonoids and phenylpropanoids, unique naphthodianthrones, including hypericin and hyperforin, which exhibit potent antimicrobial and anti-inflammatory properties [94]. Several studies in animal burn wound models have demonstrated the superior effects of *H. perforatum*-based treatments compared to standard therapies, showing enhanced tissue granulation, epithelialization, angiogenesis, and inflammation control compared to topical 10% povidone−iodine treatment [95] or SSD cream [96].

*Camellia sinensis* (*green tea*) is a natural remedy rich in polyphenols, particularly catechin-type flavonoids, which have shown promise in treating various skin disorders, including wounds and burns [97]. Green tea polyphenols are commercially available in highly concentrated and purified forms and have been shown to exhibit antimicrobial and anti-inflammatory properties. Their mechanisms of action include the reduction of proinflammatory cytokine levels (TNF-α, IL-1β, and IL-6) and inhibition of neutrophil infiltration in the affected area. In addition, they promote vascularization by stimulating VEGF- A production and contribute to proper tissue remodeling [98]. A recent clinical trial evaluating a cream containing 10% hot water green tea extract for the treatment of second-degree burns found its efficacy to be comparable to that of the standard SSD cream. Furthermore, the extract exhibited significant antimicrobial activity, inhibiting the growth of *P. aeruginosa*, *S. aureus*, and *E. coli* [99].

*Curcumin* is a dimeric derivative of ferulic acid produced by *Curcuma longa* (turmeric) and is a polyphenolic pigment responsible for the characteristic yellow color of turmeric rhizomes [100]. It has garnered increasing attention in medicinal research due to its diverse pharmacological activities and potential applications in the treatment of various conditions [101]. In burn wound management, curcumin may have dual benefits by promoting wound healing and alleviating local pain [102,103,104]. In addition to its well-documented antimicrobial and anti-inflammatory properties, curcumin facilitates multiple wound-healing processes, including fibroblast proliferation, collagen deposition, granulation tissue formation, epithelialization, and neo-angiogenesis, without inducing keloid formation, as demonstrated in several burn wound animal model studies [102,105,106,107]. Specifically, curcumin has been shown to be effective at concentrations as low as 0.1%, where it induces a less pronounced inflammatory response and enhances epithelialization compared to standard SSD cream in a rat burn wound model [108]. In addition, curcumin exhibits anti-biofilm activity against wound-infecting pathogens, such as *P. aeruginosa*, further supporting its potential therapeutic application in burn treatment.

*Essential oils* have been widely studied for their antimicrobial and therapeutic properties, primarily due to the presence of monoterpenes, which exhibit potent antibacterial and antifungal activities. However, clinical data supporting the potential benefits of these treatments in burn therapy are limited. In a case study, two patients with burn wounds received standard care, with one patient additionally treated with a mixture of essential oils, including lavender and eucalyptus oils. The patient who received only standard treatment developed complications, including sepsis caused by two bacterial strains, whereas the patient who was additionally treated with essential oils did not. Even though this case study suggests a potential protective effect, further research is needed to establish clinical efficacy, as essential oils have demonstrated strong antimicrobial properties in in vitro studies [109]. In addition, essential oils may have therapeutic applications beyond their antimicrobial activity. A meta-analysis of multiple clinical studies suggests that aromatherapy with essential oils may help alleviate pain and anxiety in patients with burns, further supporting their potential role in burn care [110].

Many other traditional plants and products, such as *Plantago major* [111], wheat bran [112], longan seeds [98], and birch [113], have been studied for their potentially beneficial effects in burn healing and were found to be effective, at least in animal models. However, there are only a limited number of studies, and further evidence is required.

### 4.4. Antimicrobial Peptides

Antimicrobial peptides (AMPs), also known as host defense peptides, are small (typically composed of 10–60 amino acids), naturally occurring proteins that play a crucial role in the innate immune system by defending the host against a broad spectrum of microorganisms, including bacteria, fungi, viruses, and even some parasites. They are produced by various organisms, including humans, animals, plants, and bacteria. In recent years, AMPs have gained significant attention as new generation of antimicrobial agents, leading to the deposition of more than 3500 AMPs in the Antimicrobial Peptide Database (APD; http://aps.unmc.edu/AP/, accessed on 31 January 2024), out of which 67.6% are derived from animals, 10.5% from bacteria, 8.6% are synthetic, 6.9% from plants, 5.2% from predicted organisms, and a small number from other sources.

Their primary mode of action involves the disruption of microbial cell membranes through electrostatic interactions. Specifically, positively charged AMPs interact with negatively charged bacterial membranes, allowing them to either form channels or transmembrane pores, leading to ion leakage and loss of essential biomolecules or acting in a detergent-like manner to disintegrate the membrane. Additionally, some AMPs penetrate bacterial cells and inhibit DNA/RNA or protein synthesis, while others stimulate the immune system, promoting wound healing and reducing inflammation [114,115]. Although AMPs are found in diverse living organisms, their low natural abundance and small molecular weight make the isolation and purification of AMPs from natural sources challenging [116]. Instead, these peptides are often produced in vitro using recombinant DNA technology, solid-phase peptide synthesis, or enzymatic hydrolysis of precursor proteins using microbial proteases. Recent eco-friendly approaches have explored the use of protein-rich waste materials from the agri-food sector as cost-effective substrates for the production of bioactive peptides [117,118].

Currently, several bioactive peptides are used in clinical settings as a last-resort treatment against multidrug-resistant Gram-positive (bacitracin, gramicidin) and Gram-negative bacteria (polymyxins). However, the therapeutic application of AMPs faces significant challenges due to their susceptibility to enzymatic degradation, potential cytotoxicity, and the need for optimization to enhance their stability and efficacy. Infected tissues often exhibit high proteolytic activity, driven by both bacterial and human defense cell protease activity. Consequently, unless an AMP is specifically designed for proteolytic stability, it may be rapidly degraded in situ, resulting in a loss of antimicrobial effectiveness [119]. This challenge can potentially be addressed through the development of efficient delivery systems that enable the sustained release of AMPs, which may improve their stability, reduce toxicity, and enhance their effectiveness through local in situ release [119]. For instance, a combination of anionic polymers, like alginate, with cationic AMPs obtained from whey protein via enzymatic hydrolysis was shown to form a stable delivery system with no observed decrease in bioactivity [120,121]. In addition, cathelicidin-DM, a bioactive peptide immobilized in a chitosan-based hydrogel carrier, exhibited beneficial activity in *S. aureus*-infected and non-infected full-thickness wounds in a mouse model with respect to hemostatic properties and enhanced healing [122]. Moreover, a composite nanoparticle made of poly (lactic-co-glycolic acid) and polytannic acid, coated with AMP Dermaseptin-PP, was found to be stable and to significantly accelerate wound healing while inhibiting microbial growth in in vivo studies [123].

### 4.5. Probiotics

Probiotics are beneficial microorganisms that are naturally present in various locations in the human body, including the skin. These microorganisms are known to exert numerous positive effects on human health, primarily by protecting against pathogens. In response to skin injury, including burns of any degree, the diversity and abundance of the local microbiota are disrupted. Although the microbiota can gradually restore itself to unaffected areas, this process is inherently slow. However, the introduction of exogenous bacteria may lead to an imbalance between the host skin microbiota and external microorganisms, potentially impairing the wound-healing process and contributing to skin infections. Lactic acid bacteria (LAB) are among the most studied species of probiotics for wound healing, including strains such as *Lactobacillus plantarum*, *Lactobacillus fermentum*, *Lactobacillus acidophillus*, *Lactobacillus rhamnosus*, *Lactobacillus bulgaricus*, *Lactobacillus reuteri*, *Lactobacillus delbreuckii*, *Lactobacillus salivarus*, and *Bifidobacterium lactis* [124]. They are known for their ability to produce lactic acid, which serves several purposes, including acidifying the environment (reducing pH) and inhibiting the growth of pathogens. Other proven mechanisms by which probiotics exert their beneficial effects are strain-specific and include mechanisms such as (i) direct killing of pathogens by the production of bacteriocins or other antimicrobial molecules, (ii) competitive displacement of pathogenic bacteria, (iii) modulation and activation of lymphocytes, natural killer cells, and macrophages through induced production of cytokines, and (iv) activation of epithelial cells and stimulation of fibroblast proliferation and/or migration [125,126].

According to the literature [126,127,128,129,130,131,132,133], topical probiotic therapy has been explored in both animal and human models of cutaneous injury, with the primary aim of reducing infection in order to improve healing. In animal burn wound models, probiotics and kefir have been shown to reduce the production of inflammatory markers. In an infected animal wound model, probiotics were found to enhance the immune response and reduce the incidence of septicemia. In human patients with burns, topical probiotics demonstrated a reduction in bacterial load comparable to that of the standard SSD cream. In relation to biofilm formation, in vitro studies have shown that the addition of live probiotics to pathogenic bacterial cultures can inhibit biofilm development by approximately 50% [134]. Specifically, these studies have revealed that distinct species antagonism occurs within biofilms between pathogenic and “commensal” species, highlighting the importance of probiotic bacteria in biofilm formation.

Overall, topical probiotics have demonstrated efficacy in improving various aspects of wound healing in both human and animal models. However, several questions remain unanswered, including the optimal dosing regimen and the most effective and sustainable delivery methods. Most studies have employed live microorganisms applied to the wound site via gauze pads, which were routinely bandaged and replaced daily with fresh cultures. This approach, although effective, is impractical from a clinical perspective and requires further optimization to become a viable treatment option.

Therefore, in order to prepare effective probiotic wound formulations for their sustained topical delivery directly to the wound site, an adequate carrier is needed. Sustained-release systems are advantageous for long-term care as they provide a continuous “inflow” of the active substance, in this case, LAB, thus continually boosting their presence in the wound area and maximizing their anti-infective potential. Promising approaches include the encapsulation of probiotics within polymer or hydrogel matrices (e.g., collagen, pectin, chitosan, alginate, and silk fibroin) [135,136,137] or their immobilization onto suitable materials (e.g., activated charcoal) [138]. In addition, probiotic carriers can be further functionalized with other supporting agents, such as bioactive peptides [121] or prebiotics. The efficacy of prebiotic-probiotic wound dressings has been tested in vivo using an infected burn wound model in rats [136]. The results demonstrated that these formulations significantly enhanced antibacterial activity, exhibited favorable properties for promoting wound healing, and showed strong potential for the treatment of burn injuries. Table 1 clusters the most recent studies on novel wound dressings with probiotics for potential applications in wound healing.

## 5. Recent Patents and Future Perspectives

**Figure 3:**
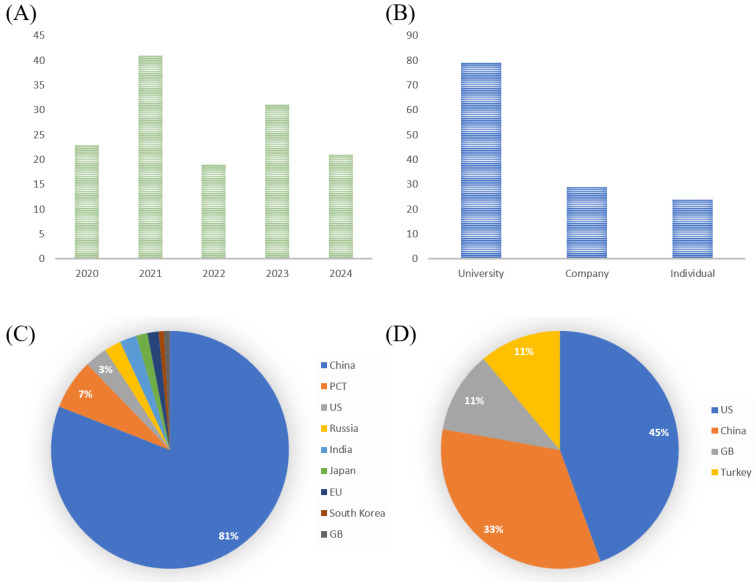
A summary of patent distribution for the period 2020–2025, presented according to (**A**) publication year, (**B**) applicant type, (**C**) applicant country, and (**D**) distribution of PCT applicant. Data were collected from the WIPO Patentscope database (https://patentscope.wipo.int) using the search terms “wound dressings AND burn wound” (accessed on 28 February 2025).

Although research is generally advancing rapidly, the transition from laboratory discoveries to clinical studies progresses much slower. Moreover, the path toward commercialization tends to be longer and more complex. In this context, patents often serve as a critical bridge between early-stage research and eventual market introduction of products. Analyzing patent activity may help better anticipate future trends and identify areas of growing interest. For the purpose of this review, the WIPO Patentscope database was searched using the Boolean combination “wound dressings AND burn wound” with the search finalized on 28 February 2025. This search identified 448 patents, with a particular focus on those published within the last five years (135 patents published between 2020 and 2025; Figure 3A). Notably, the number of patents filed by universities during this period was 2.7 times higher than that filed by companies, supporting the observation of research and commercialization trends (Figure 3B). When analyzing the geographical distribution of patent applicants, China holds a clear lead, highlighting its strategic focus on innovation-driven technologies (Figure 3C,D).

Table 2 presents selected examples of relevant patents.

The analysis of recent patents reflects an ongoing preference for well-established metallic antimicrobial agents, particularly silver in its various forms, including ionic silver (e.g., [174]), silver compounds such as SSD (e.g., [169]), and silver nanoparticles (e.g., [163]). Other frequently employed metal-based antimicrobials include zinc oxide (e.g., [168]), copper sulfide (e.g., [168]), ferrous sulfate (e.g., [154]), and bismuth-based compounds (e.g., [148]), due to their broad-spectrum antimicrobial properties. While these agents have demonstrated efficacy in reducing microbial load, they mostly do not adequately address the complexities of the regenerative aspects of wound healing.

Conversely, there has been growing interest in incorporating natural bioactive compounds into wound dressing formulations. Agents such as *Coptis chinensis* (e.g., [177]), curcumin (e.g., [149]), fungi-derived beta-glucans (e.g., [150]), and honey (e.g., [153]) offer multifunctional benefits, combining or providing antimicrobial activity with antioxidant, anti-inflammatory, and pro-healing properties. Moreover, relatively recent innovations in burn wound management have considered bioinspired solutions, which include the integration of extracellular vesicles (e.g., [152]), antimicrobial peptides (e.g., [159]), fibroblast growth factors (e.g., [155]), and probiotics (e.g., [157]) into wound dressings. These approaches aim to actively modulate the wound microenvironment, promoting tissue regeneration while simultaneously inhibiting pathogenic colonization and biofilm development.

Given the unique challenges associated with burn wound healing, including high susceptibility to infection, the need for rapid tissue regeneration, and minimal scarring—future strategies are expected to focus on achieving synergistic therapeutic effects. This will likely involve the controlled and sustained delivery of multiple bioactive agents from polymeric matrices that not only serve as carriers for drug delivery but also actively support the healing process. One example is the intensive application of chitosan, which has demonstrated antimicrobial activity and has been widely used in advanced formulations over the past five years (e.g., [147,150,152,156,161,165,166,170,175]). This further suggests a leading trend that efficient burn management requires multifunctional and synergistic wound dressings.

Finally, alternative strategies further expand the potential for innovation in burn wound care. One promising approach involves the development of responsive dressings, particularly those that are pH-sensitive (e.g., [178]), which can detect early signs of bacterial infection and provide timely feedback for clinical intervention. Another interesting approach focuses on dressings designed to rapidly absorb heat from the burn site [180], thereby decreasing thermal injury, preventing further tissue damage, and reducing the risk of infection. Importantly, these advanced systems are not isolated solutions but can be integrated with multifunctional wound dressings that deliver bioactive agents in a controlled and sustained manner while optimizing moist wound environments. This holistic approach has the potential to address the complex and multifactorial nature of burn wound healing, offering comprehensive solutions that can improve clinical outcomes. Thus, interdisciplinary collaboration between materials scientists, microbiologists, engineers, and clinical practitioners will be crucial in driving these innovations. Addressing regulatory and manufacturing challenges is also key to ensuring the widespread adoption of these advanced therapies. As burn wounds continue to pose significant clinical and economic burdens worldwide, the pursuit of innovative, sustainable, and cost-justified solutions remains a priority.

## Figures and Tables

**Table 1:** In vivo and in vitro studies highlighting the novel wound dressing with probiotics in the last 5 years.

Type of Study	Polymer	Probiotic Strain (With/Without Prebiotic)	Pathogen Strain	Ref.
In vitro and in vivo (Wistar rat model)	gelatin, HA, and LRHA hydrogel	*L. reuteri*	*E. coli* *S. aureus* *Salmonella* spp.	[139]
In vitro and in vivo (mice model)	oxidized *Bletilla striata*-chitosan composite hydrogel	*L. plantarum*	*E. coli* *S. aureus* *P. aeruginosa*	[140]
In vitro and in vivo (mice model)	hyaluronate-adipic dihydrazide/aldehyde-terminated Pluronic F127/fucoidan hydrogel	*L. rhamnosus*	multi-resistant *P. aeruginosa*	[141]
In vitro and in vivo animal model	pectin, alginate, and chitosan	*L. plantarum* (ATCC 1058), fructooligosaccharide (FOS)	*P. aeruginosa* (ATCC 9027) *S. aureus* (ATCC 6538)	[136]
In vitro and in vivo animal model	Guar gum and PVA	*L. plantarum*	*P. aeruginosa*	[142]
In vivo animal model	Silk fibroin/sodium alginate	*L. casei*	*E. coli* *S. aureus*	[137]
In vitro and in vivo (Wistar rat model)	Probiotic hydrogels	*L. paracasei* (TYM202), extracellular polysaccharides 9EPS0 from *B. velezensis* (M76T11B)	*E. coli* *S. aureus*	[143]
In vivo (rat model)	Hybrid bilayer wound dressing	*L. brevis* (KCTC 3498)	*S. aureus subsp. aureus* KCCM 40050	[144]
In vivo (mice model)	Sponge dressings	*L. plantarum* UBLP-40 (MTCC 5380)	*S. aureus* 9144	[145]

**Table 2:** Selected examples of relevant patents for the period 2020–2025 generated from the WIPO Patentscope database.

Patent Title	Composition/Key Features	Ref.
Wound dressings	An amorphous gel composed of a nitrite layer with nitrite salts and an acid layer with at least one acid, along with a solid conjugate base to create a buffer system with a pH of 3.8 to 6.0; the dressing generates nitric oxide through the acidification of the nitrite salts	[146]
Dressings and methods for wound healing	3D-printed wound dressing comprising a hydrogel matrix (alginate, gelatin, gelMA, cellulose, or chitosan) with up to 50 *w*/*v* % bioactive borate glass (BBG) containing boron	[147]
Bismuth-containing burn dressing and preparation method thereof	Bismuth-containing burn dressing with three layers: a back paste layer, an antibacterial matrix layer made of a bismuth-infused polyurethane sponge, and a releasing layer	[148]
Nanofiber dressing	A core-shell structure drug-loaded nanofiber burn wound dressing, prepared by electrostatic spinning using polylactic acid (PLA), 2-hydroxypropyl-alpha-cyclodextrin (HP-alpha-CD), and curcumin (12.4–13.4%)	[149]
Dressing containing polysaccharides, as well as preparation method and application of the dressing	*Ganoderma lucidum* beta-glucan, *Ganoderma lucidum* chitosan, glycerol, and carbomer	[150]
Production process of polyethylene glycol paste dressing	Polyethylene glycol paste dressing incorporating beta-glucose, magnolia flower extract, and *Herba houttuyniae* extract in the water phase, and sesame oil with *Melaleuca alternifolia* essential oil in the oil phase	[151]
A 3D biocompatible matrix and its uses in wound management	A 3D scaffold based on natural (e.g., chitosan, collagen, alginate) or synthetic polymers (e.g., PLA, PGA, PLGA, polysiloxanes), incorporating non-opioid analgesics, extracellular vesicles (preferably human-derived), or artificial lipid vesicles (20–150 nm in size)	[152]
Antimicrobial superabsorbent compositions	A powder containing an enzyme that converts a substrate (e.g., honey) to release hydrogen peroxide, a precursor-substrate or substrate for the enzyme, and a superabsorbent component; forming a gel upon contact with water	[153]
Use of ferrous ions in the preparation of burn infection treatment drugs and burn care products	A ferrous compound (e.g., ferrous sulfate), a protective agent, a dressing matrix (such as sodium alginate or hyaluronic acid), and a solvent; these components are mixed and heated to form a ferrous compound hydrogel, which addresses burn infections caused by *Pseudomonas aeruginosa*, reduces antibiotic resistance risks	[154]
Bio-drug for treating large-area burns, engineering skin substitutes, and large-area burn treatment dressing	Dressing composed of eupatorin, fibroblast growth factors, curcumin, *mulberry* leaf extract, *Ranunculus* polysaccharide extract, and sulfadiazine silver for large-area burn wounds	[155]
Preparation method of multifunctional double-layer heterogeneous hydrogel dressing	Multifunctional double-layer heterogeneous hydrogel dressing for large-area burn wound treatment prepared using acrylamide, N-isopropylacrylamide, chitosan, sodium alginate, polyvinyl alcohol, an antibacterial agent, and a growth factor, cross-linked by ultraviolet light	[156]
Prevention and/or treatment of wound infection	A probiotic composition for treating and/or preventing wound infections, containing *Cutibacterium acnes* cells, cellular contents, cell-free supernatant, or bioactive components derived from the supernatant; it can be formulated as an ointment, gel, or cream for application to burns or skin wounds	[157]
Nitric oxide dressing capable of directionally draining exudate, as well as the preparation process and application of nitric oxide dressing	Multilayer dressing containing nitric oxide and an activating agent	[158]
Hydrogel attached with antibacterial coating and its application	A macroporous polysaccharide hydrogel with an antibacterial coating of catechol compounds and antibacterial peptides	[159]
Method of treating borderline dermal skin burns by applying a gel of rarely cross-linked acrylic polymers with a complex of natural antimicrobial peptides FLIP7	An antimicrobial gel made from cross-linked natural acrylic polymers carrying FLIP7 natural peptides	[160]
Drug-loaded liposome hydrogel, as well as preparation and application thereof	A drug-loaded liposome hydrogel, composed of a carboxymethyl chitosan-sodium alginate hydrogel cross-linked with madecassoside and coated with liposomes carrying acetylshikonin and aloe-emodin, enabling slow, continuous, and sequential drug release	[161]
Curcumin-containing polymer and application thereof in promoting healing of burns	ε-poly-*L*-lysine and γ-polyglutamic polymer matrix with curcumin	[162]
Fabrication of green silver nanoparticle-embedded microsphere and therapeutic activity against bacteria-infected burn and excision wound	Green silver nanoparticle-embedded mucilage microspheres with high water absorption capacity	[163]
Antibacterial dressing for burn wounds	An antibacterial burn wound dressing made of nano-silver-coated gauze and polyurethane foam connected to a negative pressure drainage device	[164]
Preparation method of silk-spider silk composite silk fibroin nano-microspheres containing chitosan-modified graphene oxide	Chitosan-modified graphene oxide silk-spider silk composite fibroin nanospheres with excellent biocompatibility and biodegradability	[165]
Composition for preparing burn and wound dressing, as well as preparation and preparation method thereof	A composite burn wound dressing made from silk fibroin (as an antioxidant), active iodine (for broad-spectrum antibacterial action), and a chitosan-based water-absorbing framework, resulting in a porous, asymmetric dressing	[166]
Composition for treating wounds or burns comprising aged mink oil by the addition of snake venom as the active ingredient	A composition for treating wounds and burns and promoting skin cell regeneration, using aged mink oil combined with snake venom as the active ingredient	[167]
Alginate-encapsulated bacterial cellulose composite photo-thermal antibacterial medical dressing and preparation method thereof	A composite photo-thermal antibacterial medical dressing made of an inner bacterial cellulose/zinc oxide/copper sulfide porous membrane and an outer alginate encapsulation layer	[168]
Preparation and antibacterial property research of silver sulfadiazine spray	A silver sulfadiazine spray formulated with a surfactant and polymer, offering a non-greasy, evenly distributed protective film that improves patient comfort and compliance	[169]
Double-sustained-release drug-loaded hydrogel dressing with semi-interpenetrating network entrapped double-layer microspheres, as well as preparation method and application thereof	A dual pH- and temperature-sensitive hydrogel incorporates double-layer microspheres (calcium alginate core with bovine serum albumin and a chitosan shell with azithromycin) within a hydrogel matrix containing acrylic acid, methacrylic acid 2-ethyl ester, and oligomeric (ethylene glycol) methyl ether methacrylate, with embedded gentamicin sulfate	[170]
Preparation method and application of drug-loaded hydrogel	Drug-loaded injectable hydrogel composed of oxidized dextran (Odex) and gelatin grafted with protocatechuic acid (GT-PCA), prepared by mixing both solutions and cross-linking through a Schiff base reaction to form the GT-PCA/Odex hydrogel	[171]
Traditional Chinese medicine composition for treating burn and scald wounds, traditional Chinese medicine preparation, and preparation method	Traditional medicine composition for treating burn and scald wounds, made from *Astragalus membranaceus*, *Rheum officinale*, *Angelica sinensis*, *Carthamus tinctorius* (safflower), *Rhizoma sparganii*, *Curcuma zedoaria*, *Cercis chinensis* (Chinese redbud bark), *Angelica dahurica* (Dahurian angelica root), *Saposhnikovia divaricata* (radix saposhnikoviae), and *Endothelium corneum gigeriae galli*	[172]
Smart-DRE-M: biopolymer composite-based nano-fibrous wound dressing material	Core-shell nanofiber wound dressing fabricated from natural, low-cost biomaterials, including mucilage/gum from *Cochlospermum gossypium* (Yellow Silk Cotton Tree) and *Canthium coromandelicum* (Native Indian Herb), along with leaf extract of *Chromolaena odorata* (Siam weed), fluorine-doped carbon dots loaded with the anti-inflammatory drug Zaltoprofen and taurine as a bio-piezoelectric component	[173]
Improved biosynthetic wound and burn dressing with silver-based broad antimicrobial activity	Dressing with silver ion antimicrobial coating	[174]
Preparation method of hemostatic and antibacterial dressing containing field thistle herb extract	Hemostatic and antibacterial dressing containing field thistle herb extract, chitosan, growth factors, and hyaluronic acid	[175]
Preparation and application of antibacterial-modified exosome burn wound healing-promoting biological dressing	Antibacterial biological dressing composed of chitosan porous material loaded with artificially modified exosomes carrying broad-spectrum antibacterial agents	[176]
Coptis chinensis skin healing paste and clinical research method for treating burns by using gauze strips of paste	Skin healing paste (composed of *Coptis chinensis*, *Angelica sinensis*, *Phellodendron chinense* Schneid, *Radix Rehmanniae Recens*, *Rhizoma Wenyujin Concisum*, raw *Sanguisorba officinalis*, frankincense, myrrh, raw rhubarb, beeswax, and sesame oil) is soaked onto multiple layers of gauze strips	[177]
pH Indicator dressing for monitoring of wound and infection	A biocompatible polymer integrated with a pH-indicating agent, applied as a fiber, hydrogel, or microsphere on the wound site, enables early detection of infection or chronic wound states by color change, supporting monitoring of acute, chronic, or burn wound healing	[178]
Polyurethane foam dressing material containing silver-activated carbon composite and producing method thereof	Three-layered antibacterial polyurethane foam dressing composed of a microporous polyurethane foam layer loaded with a silver-activated carbon composite, a moisture-permeable and waterproof layer, and a perforated polyurethane film layer; it provides consistent, stable release of antimicrobial silver particles regardless of exudate levels, promoting effective wound healing and protection against infection	[179]

## Data Availability

The original contributions presented in this study are included in this article; further inquiries can be directed to the corresponding author.
